# Pheochromocytoma-related cardiomyopathy presenting as acute myocardial infarction: A case report

**DOI:** 10.1097/MD.0000000000024984

**Published:** 2021-03-19

**Authors:** Xuandong Jiang, Weimin Zhang, Qiang Fang

**Affiliations:** a Intensive Care Unit, The First Affiliated Hospital of Zhejiang University School of Medicine, Hangzhou; b Intensive Care Unit, Dongyang People's Hospital, Dongyang, Zhejiang, PR China.

**Keywords:** acute myocardial infarction, catecholamine cardiomyopathy, pheochromocytoma, takotsubo cardiomyopathy

## Abstract

**Introduction:**

Pheochromocytoma (PHEO)-related cardiomyopathy is a rare condition in which release of a large amount of catecholamines leads to severe vasoconstriction, coronary vasospasm, myocardial ischemia, injury, and necrosis. Its clinical manifestations can be similar to those of acute coronary syndrome.

**Patient concerns:**

A 63-year-old woman was diagnosed with acute non-ST segment elevation myocardial infarction following chest pain for 8 hours. The results of coronary angiography were normal. The patient developed dyspnea, cough with frothy pink sputum, paroxysmal sweating, arrhythmia, and blood pressure fluctuation, and was transferred to the intensive care unit for monitoring and treatment.

**Diagnosis:**

PHEO, catecholamine cardiomyopathy (CICMP)

**Intervention:**

After monitoring the pulse index continuous cardiac output and treatment with α and β adrenergic receptor blockers for 18 days, laparoscopic resection of the left adrenal mass was performed.

**Outcomes:**

The patient's condition improved and she was discharged 31 days after admission. Outpatient follow-up examinations 1 month and 1 year later did not show recurrence.

**Lessons:**

PHEO can cause CICMP, the manifestations of which are partly similar to those of takotsubo cardiomyopathy (TTC). Once the patient's condition stabilizes, surgery should be considered. Fluid management is necessary, and agents such as α and β adrenergic receptor blockers should be administered.

## 1 Introduction

Acute catecholamine cardiomyopathy (CICMP) is a rare and life-threatening complication that occurs in about 10% of patients with pheochromocytoma (PHEO) or paraganglioma.^[1]^ It has 3 common types:^[2]^ takotsubo cardiomyopathy (TTC), dilated cardiomyopathy, and hypertrophic cardiomyopathy. It is characterized by the release of large amounts of catecholamines including norepinephrine and epinephrine. When in excess, these catecholamines can induce severe vasoconstriction, coronary vasospasm, myocardial ischemia, injury, and necrosis, with various clinical manifestations. In severe cases, secondary pulmonary edema, cardiogenic shock, and even cardiac arrest might occur. CICMP increases the risk of mortality, has a poor prognosis, and often requires surgical intervention. Therefore, early diagnosis and management are critical.

We report here the case of a 63-year-old female patient with acute myocardial infarction. On investigation, PHEO was detected; the cardiac ultrasound was similar to that seen in TTC, and she was diagnosed with acute CICMP.

## 2 Case report

This study adhered to the tenets of the Declaration of Helsinki. The patient provided informed consent for the publication of this case report and any associated images.

A 63-year-old woman was admitted to the hospital on July 22, 2019 owing to chest pain for 8 hours. She had a more than 10-year history of hypertension; her highest recorded blood pressure was 200 mm Hg. She was on irbesartan 150 mg qd, captopril 25 mg bid, and nifedipine sustained-release tablets 20 mg bid to lower her blood pressure; however, her blood pressure was not regularly monitored. She underwent a total hysterectomy 18 years ago for a hysteromyoma, and the postoperative recovery was fair. She had a history of penicillin allergy.

When admitted to the emergency room, her heart rate was 93 bpm, her blood pressure was 137/102 mm Hg, and her high-sensitivity troponin T and pro-B-type natriuretic peptide levels were 2.53 ng/ml and 464.6 pg/ml, respectively. Emergency myocardial enzyme levels were as follows: aspartate aminotransferase: 97 U/L; creatine kinase: 565 U/L; creatine kinase isoenzyme: 97 U/L; and lactate dehydrogenase: 536 U/L; the other parameters were normal. Color Doppler echocardiography showed segmental ventricular wall motion abnormalities, aortic regurgitation (mild), mitral regurgitation (mild), tricuspid regurgitation (moderate), left atrial enlargement, pulmonary hypertension (mild), aortic arteriosclerosis, and an ejection fraction of 56%. An electrocardiogram showed sinus rhythm, premature atrial contractions with aberrant ventricular conduction, and premature atrial contractions in couplets.

The patient was diagnosed with acute non-ST segment elevation myocardial infarction. Aspirin and Plavix (clopidogrel sulfate) were administered to inhibit platelet aggregation, Lipitor (atorvastatin calcium) was administered to stabilize the plaques, and emergency coronary angiography was performed. During the surgery, no significant stenosis was found in the right coronary artery, left main coronary artery, or circumflex artery; however, the proximal segment of the anterior descending branch had 20% stenosis. After the surgery, the patient had difficulty breathing, paroxysmal sweating, cold limbs, cough with frothy pink sputum, 90% fingertip blood oxygen saturation under face mask oxygen inhalation, and wet rales in both lungs. She was transferred to the intensive care unit (ICU) for monitoring and treatment. Endotracheal intubation was performed immediately, and she was put on a ventilator to assist breathing; norepinephrine was administered to maintain the blood pressure. However, the blood pressure fluctuated significantly, and the average arterial pressure was between 50 and 100 mm Hg.

**Figure 1:**
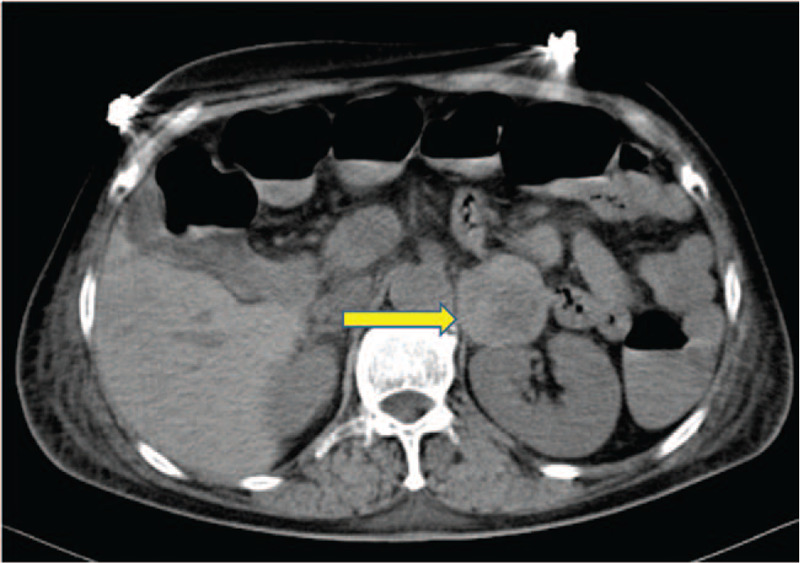
Computed tomographic scan demonstrating a left adrenal mass (arrow).

Indicative of segmental ventricular wall abnormalities, color Doppler echocardiography reexamination on July 23 revealed significantly reduced motion in the ventricular septum, the basal segment to the apical segment of the anterior left ventricular wall, and the middle segment to the apical segment of the inferior left ventricular wall. The left atrium and left ventricle were enlarged, with left heart failure, aortic regurgitation (mild), mitral regurgitation (mild), and aortic arteriosclerosis also apparent. The ejection was 38%. TTC was suspected; hence, pulse indicator continuous cardiac output (PICCO) was measured to calculate the hemodynamics. The results were as follows: cardiac index: 2.16 L/minutes/m^2^; intrathoracic blood volume index: 1206 ml/minutes/m^2^; global end-diastolic volume index: 965 mL/m^2^; and extravascular lung water index: 16.4 ml/kg. Computed tomography detected a left adrenal mass (Fig. 1). Laboratory investigations revealed a free normetanephrine level of 8900.0 pg/ml (normal value: 145 pg/ml) and a free metanephrine level of 1030.0 pg/ml (normal value: 62 pg/ml). The patient was diagnosed with PHEO.

**Figure 2:**
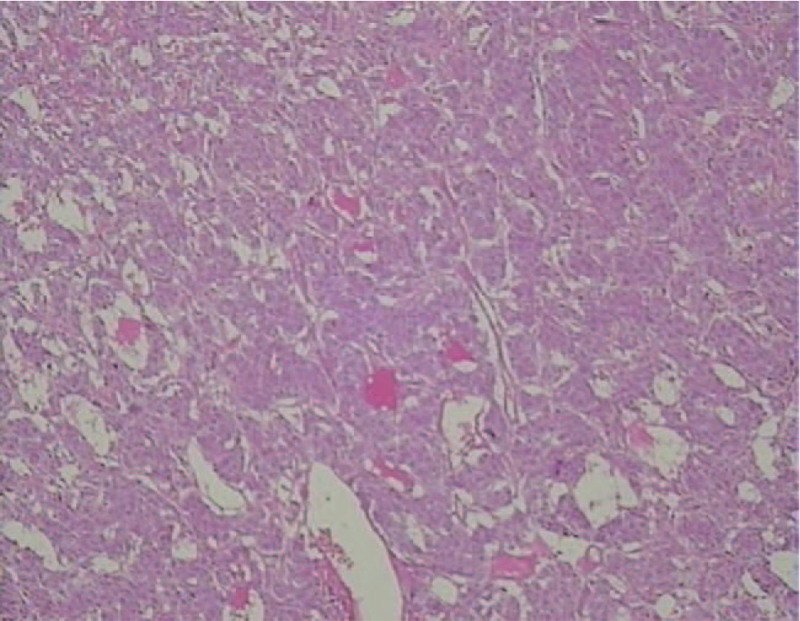
Light micrograph of a hematoxylin-and eosin-stained section (100×). The tumor cells are relatively large and polygonal with bicolor cytoplasm, and are nested or alveolar and separated by vascular fibers to form organ-like structures.

Milrinone was administered to strengthen the heart, and phentolamine was administered to control the blood pressure. After the necessary preoperative preparation, laparoscopic left adrenal mass resection was performed on August 9 once the hemodynamics became relatively stable. During the surgery, the left adrenal gland and tumor were observed; the tumor size was about 4 × 4 × 3.6 cm. Microscopic examination of hematoxylin- and eosin-stained sections showed that the tumor cells were relatively large in size and polygonal in shape, with bicolor cytoplasm; they were nested or alveolar and separated by vascular fibers to form organ-like structures (Fig. 2).

After the surgery, the patient continued to be monitored in the ICU and was soon weaned from mechanical ventilation. However, she still had paroxysmal arrhythmia and hypotension, which improved after esmolol was administered. She was transferred to the Department of Urological Surgery on August 15 and discharged from the hospital on August 21. At the outpatient follow-ups 1 month and 1 year later, PHEO had not recurred, blood pressure was well-controlled, and cardiac function was normal.

## 3 Discussion

PHEO and CICMP are rare diseases, with only a few cases presenting with acute coronary syndrome reported previously.^[3]^ The patient in our study developed chest pain and had increased levels of myocardial injury markers, with segmental ventricular wall motion abnormalities on color Doppler echocardiography. She was diagnosed with acute non-ST segment elevation myocardial infarction in the Emergency Department. However, coronary angiography showed no acute occlusion of the coronary arteries, which can result from catecholamine-induced reversible coronary artery spasms and lead to myocardial ischemic and hypoxic injury.^[2]^

Many factors are involved in the pathogenesis of CICMP, the most prominent of which is calcium-mediated cardiomyocyte injury. Furthermore, catecholamines and their oxidation products have been proven to have myocardial toxicity.^[4]^ By increasing the permeability of the muscle cell membrane, they induce intracellular calcium influx to produce irreversible myocardial ischemia, followed by injury and necrosis. Thus, a series of clinical features are seen, including hypertension, dilated cardiomyopathy, hypertrophic cardiomyopathy, pulmonary edema, cardiogenic shock, and TTC.

Our patient's cardiac ultrasonographic features were similar to those in TTC, including impaired motor function in the middle and apical segments of the left ventricle.^[5]^ PHEO-induced Inverted-TTC has been reviewed,^[6]^ the motion in the basal segment was decreased; however, the function of the apical segment was maintained. Research has shown that cardiac magnetic resonance imaging can identify the type of catecholamine-induced myocarditis,^[7]^ making it a valuable noninvasive diagnostic method for evaluating CICMP.

Currently, the understanding of CICMP is relatively inadequate, and guidelines on CICMP management are lacking. Stress-induced cardiomyopathy is usually self-limiting, during which patients have unstable hemodynamics, paroxysmal arrhythmia, and fluctuating blood pressure, which might require the support of non-adrenal positive inotropic drugs, such as milrinone. Cardiogenic shock can occur in severe cases and requires short-term use of mechanical circulatory assist devices. Successful treatment of patients via intra-aortic balloon pump counter pulsation and extracorporeal membrane oxygenation has been reported.^[8,9]^ Our patient was not diagnosed with CICMP in the early stage; hence, she was administered norepinephrine to maintain her blood pressure, which did not improve her condition. It is important to identify and treat such patients as early as possible for a favorable prognosis.

The 2014 Clinical Practice Guidelines of the Endocrinology Society recommend surgical treatment for PHEO.^[10]^ PHEO resection is a high-risk surgery, and adequate preoperative preparations are crucial. Use of α and β adrenergic blockers, as well as calcium channel blockers, is recommended. It has been reported that α and β adrenergic blockers can suppress sympathetic activity and thus reduce surgical mortality.^[11]^ It is recommended that treatment with β adrenergic blockers be initiated slowly after the administration of α adrenergic blockers. Our patient received adequate rehydration therapy and phentolamine and esmolol before resection of the adrenal mass; nevertheless, she developed hypotension after resection. After postoperative esmolol treatment, her blood pressure stabilized.

Perioperative monitoring of fluid is vital for patients with PHEO. In the early stage, patients suffer from cardiomyopathy, the heart function is suppressed, and pulmonary edema and fluid overload is likely to occur. However, after the acute stage, the heart function improves significantly, and fluid insufficiency might occur, leading to blood pressure fluctuation. Our patient was monitored using PICCO, which confirmed that vasoactive drugs could be discontinued after fluid supplementation. Fluid preparation before surgery is necessary. In a study of 123 PHEO patients who underwent laparoscopic adrenalectomy, 54 patients (43.9%) developed hypotension;^[12]^ hence, fluid management is crucial. If PICCO monitoring is not possible, use of a central venous catheter to monitor the central venous pressure is recommended.

## 4 Conclusion

When acute coronary syndrome without coronary artery occlusion occurs, CICMP should be considered. CICMP can manifest as TTC, which is a self-limiting disease, and might require support with non-adrenergic positive inotropic drugs. If PHEO is diagnosed, surgical treatment is recommended, and adequate preparation should be performed before the operation. Hypotension might occur after the surgery, and support treatment such as fluid management should be adequately provided.

## Acknowledgments

We would like to thank Editage (www.editage.cn) for English language editing.

## Author contributions

**Conceptualization:** Weimin Zhang, Qiang Fang.

**Data curation:** Xuandong Jiang.

**Supervision:** Weimin Zhang.

**Writing – original draft:** Xuandong Jiang.

**Writing – review & editing:** Xuandong Jiang, Weimin Zhang, Qiang Fang.
